# The Effectiveness of Artificial Intelligence Conversational Agents in Health Care: Systematic Review

**DOI:** 10.2196/20346

**Published:** 2020-10-22

**Authors:** Madison Milne-Ives, Caroline de Cock, Ernest Lim, Melissa Harper Shehadeh, Nick de Pennington, Guy Mole, Eduardo Normando, Edward Meinert

**Affiliations:** 1 Digitally Enabled PrevenTative Health Research Group Department of Paediatrics University of Oxford Oxford United Kingdom; 2 Imperial College Healthcare NHS Trust London United Kingdom; 3 Ufonia Limited Oxford United Kingdom; 4 Institute of Global Health University of Geneva Geneva Switzerland; 5 Oxford University Hospitals NHS Foundation Trust Oxford United Kingdom; 6 Department of Primary Care and Public Health Imperial College London London United Kingdom; 7 Centre for Health Technology University of Plymouth Plymouth United Kingdom

**Keywords:** artificial intelligence, avatar, chatbot, conversational agent, digital health, intelligent assistant, speech recognition software, virtual assistant, virtual coach, virtual health care, virtual nursing, voice recognition software

## Abstract

**Background:**

The high demand for health care services and the growing capability of artificial intelligence have led to the development of conversational agents designed to support a variety of health-related activities, including behavior change, treatment support, health monitoring, training, triage, and screening support. Automation of these tasks could free clinicians to focus on more complex work and increase the accessibility to health care services for the public. An overarching assessment of the acceptability, usability, and effectiveness of these agents in health care is needed to collate the evidence so that future development can target areas for improvement and potential for sustainable adoption.

**Objective:**

This systematic review aims to assess the effectiveness and usability of conversational agents in health care and identify the elements that users like and dislike to inform future research and development of these agents.

**Methods:**

PubMed, Medline (Ovid), EMBASE (Excerpta Medica dataBASE), CINAHL (Cumulative Index to Nursing and Allied Health Literature), Web of Science, and the Association for Computing Machinery Digital Library were systematically searched for articles published since 2008 that evaluated unconstrained natural language processing conversational agents used in health care. EndNote (version X9, Clarivate Analytics) reference management software was used for initial screening, and full-text screening was conducted by 1 reviewer. Data were extracted, and the risk of bias was assessed by one reviewer and validated by another.

**Results:**

A total of 31 studies were selected and included a variety of conversational agents, including 14 chatbots (2 of which were voice chatbots), 6 embodied conversational agents (3 of which were interactive voice response calls, virtual patients, and speech recognition screening systems), 1 contextual question-answering agent, and 1 voice recognition triage system. Overall, the evidence reported was mostly positive or mixed. Usability and satisfaction performed well (27/30 and 26/31), and positive or mixed effectiveness was found in three-quarters of the studies (23/30). However, there were several limitations of the agents highlighted in specific qualitative feedback.

**Conclusions:**

The studies generally reported positive or mixed evidence for the effectiveness, usability, and satisfactoriness of the conversational agents investigated, but qualitative user perceptions were more mixed. The quality of many of the studies was limited, and improved study design and reporting are necessary to more accurately evaluate the usefulness of the agents in health care and identify key areas for improvement. Further research should also analyze the cost-effectiveness, privacy, and security of the agents.

**International Registered Report Identifier (IRRID):**

RR2-10.2196/16934

## Introduction

### Background

Conversational agents are among the many digital technologies being introduced into the health sector to address current health care challenges, such as shortages of health care providers, which reduce the availability and accessibility of health care services [1-3]. Conversational agents use artificial intelligence (AI), including machine learning (a statistical means of training models with data so that they can make predictions based on a variety of features) and natural language processing (NLP; the ability to recognize and analyze verbal and written language) to interact with humans via speech, text, or other inputs and outputs on mobile, web-based, or audio-based platforms [1,4]. Many of these agents are designed to use NLP so that users can speak or write to the agent as they would to a human. The agent can then analyze the input and respond appropriately in a conversational manner [5].

Conversational agents first emerged as a tool in health care in 1966, with the development of a virtual psychotherapist (ELIZA) that could provide predetermined answers to text-based user input [6]. In the decades since, the capabilities of NLP have significantly progressed and aided the development of more advanced AI agents. Many different types of conversational agents that use NLP have been developed, including chatbots, embodied conversational agents (ECAs), and virtual patients, and are accessible by telephone, mobile phones, computers, and many other digital platforms [7-10]. The types of input that conversational agents can receive and interpret have also expanded, with some conversational agents capable of analyzing movements, such as gestures, facial expressions, and eye movements [11,12].

Conversational agents have been developed for many different aspects of the health sector to support health care professionals and the general public. Specific uses include screening for health conditions, triage, counseling, at-home health management support, and training for health care professionals [8,13-15]. With phone, mobile, and online platforms being widely accessible, conversational agents can support populations with limited access to health care or poor health literacy [16,17]. They also have the potential to be affordably scaled up to reach large proportions of a population [3]. Due to this accessibility, conversational agents are also a promising tool for the advancement of patient-centered care and can support users’ involvement in the management of their own health [17,18]. Personalizable features have the potential to further improve usability and satisfaction, although more research is needed to evaluate their effectiveness in achieving their stated health outcomes and reducing costs and to ensure that there are no negative consequences for decision making or privacy [10].

Despite the large body of research concerning the application of conversational agents in health care, most reviews have limited their focus to a particular health area, agent type, or function [10,19-22]. Although there are a few recent systematic reviews that have examined a more comprehensive scope, they have presented an overall synthesis of the body of knowledge. One review developed a taxonomy that described the architecture and functions of conversational agents in health care and the state of the field but did not evaluate the effectiveness, usability, or implications for users [5]. Another systematic review investigated the outcome measures of the studies of conversational agents but limited the inclusion criteria to agents that used natural language input and had been tested with human participants [2]. Additionally, their initial database searches only retrieved 1531 articles, which raises the concern that some relevant articles may have been overlooked [2]. Their search was updated in February 2018, but given the rapid pace of technological development, there is a need to provide an update and expansion to these previous systematic reviews.

For conversational agents to be successful in health care, it is crucial to understand the effectiveness of current agents in achieving their intended outcomes. However, it is just as important to understand how users feel about and relate to these agents because the adoption of new health technologies depends on user perceptions (eg, whether they trust the technology, find it easy to use, and feel that privacy and data security are respected) [23]. User-identified problems will need to be addressed if conversational agents are to have a significant impact on health care, because their impact depends on people being willing to use them and preferring to use them over alternatives. The information gathered in this review identifies the current issues with conversational agents that need to be overcome and can be used to help determine which elements of the agents are most likely to be successful and useful in various aspects of health care. As conversational agents are often touted as having the potential to reduce the burden on health care resources, evaluations of the implications of the agents for improved health care provision and reduced resource demand also need to be assessed.

### Objectives

The primary objectives of this review are to describe the scope of conversational agents currently being used for health care activities (by patients, health care providers, or the general public), examine the user perceptions of these agents, and evaluate their effectiveness. We developed 3 main research questions to address these objectives. First, are the conversational agents investigated effective at achieving their intended health-related outcomes, and does the effectiveness vary depending on the type of agent? Second, how do users rate the usability and satisfactoriness of the conversational agents, and what specific elements of the agents do they like and dislike? Finally, what are the current limitations and gaps in the utility of conversational agents in health care? These objectives build on previous systematic reviews while widening the scope of included studies to update the body of knowledge on conversational agents in health care and to inform future research and development.

## Methods

### Database Search

The full methods for this review have been published in detail in a systematic review protocol [24]. The population, intervention, comparison, and outcome framework [25] was used to develop the search strategy, which was implemented following the PRISMA-P (Preferred Reporting Items for Systematic Review and Meta-Analyses Protocols) checklist [26]. No study design filter was used; any type of study was eligible for inclusion. The search strategy was finalized and tailored to different databases in consultation with a medical librarian. PubMed, Medline (Ovid), EMBASE (Excerpta Medica dataBASE), CINAHL (Cumulative Index of Nursing and Allied Health Literature), Web of Science, and the Association for Computing Machinery Digital Library databases were searched. The search terms were grouped into 3 themes (conversational agents, health application, and outcome assessment) to capture all studies that fit the key inclusion criteria: evaluating conversational agents used in health care. These themes were subsequently searched with the structure: conversational agent (MeSH OR Keywords) AND health application (MeSH OR Keywords) AND outcome assessment (MeSH OR Keywords). The full search strategy can be found in Multimedia Appendix 1. The search was completed on November 29, 2019.

### Inclusion and Exclusion Criteria

This systematic review aimed to assess conversational agents designed for health care purposes. Studies that evaluated at least 1 conversational agent were included. Studies targeting any population group, geographical location, and mental or physical health-related function (eg, screening, education, training, and self-management) were included. These broad inclusion criteria were established to enable an assessment of a wide range of applications of conversational agents. There were no restrictions on study type, as long as a conversational agent was evaluated, and intervention and observational studies such as cross-sectional surveys, cohort studies, and qualitative studies were included. Intervention studies were not required to have a specific comparator or any comparator.

During the screening process, studies of conversational agents that were not capable of interacting with human users via unconstrained NLP were excluded. These included conversational agents that only allowed users to select from predefined options or agents with prerecorded responses that did not adapt to subsequent user responses. The basis for this exclusion is that, without the capability of using NLP, computational methods and technologies are rudimentary and do not advance the aims of AI for autonomous computational agents. As many studies did not explicitly state whether the investigated agent was capable of NLP, a description in the paper of the conversational agent allowing free-text or free-speech input was used as an indicator for NLP, and these studies were included. Studies that did not report the architecture of the agent were excluded.

Due to the number of conversational agents in development and/or those that did not progress to the evaluation stages of development, studies that were solely descriptive were excluded. Furthermore, because of the pace at which conversational agents have developed over recent decades, studies were limited to those published during or after 2008. In 2008, the first iPhone was released, and it marks an increase in the prevalence and capabilities of digital technology. Only studies published in English were included to ensure accurate interpretation by the authors. Conference publications were also excluded from the review of peer-reviewed literature.

### Outcomes

The primary objective of this review was to provide an overview of the use of NLP conversational agents in health care. Therefore, the primary outcomes evaluated were the effectiveness of conversational agents in achieving their intended health-related outcomes and user perceptions of the agents (including but not limited to acceptability, usability, satisfaction, and specific qualitative feedback). Secondary outcomes included improvement in health care provision and resource implications for the health care system.

### Screening and Study Selection

All studies retrieved from the databases were stored in the reference management software EndNote (version X9, Clarivate Analytics), which automatically eliminated duplicates. Due to time constraints, the EndNote search function was used to extract relevant studies before the screening of the citations against the inclusion and exclusion criteria by 2 independent reviewers. Where duplicates or publications from the same study were identified, the more recent publication or the one with the most detail was selected for inclusion in the review. All disagreements were discussed, and if a consensus was not reached, a third reviewer was consulted. Full EndNote search details are shown in Multimedia Appendix 2.

The full texts of the articles that met the inclusion criteria were screened by one of the reviewers. Of the screened articles deemed eligible for inclusion, 58 were conference or meeting abstracts and did not have full texts available; therefore, they were excluded. This highlights the early developmental stages of many of these agents.

### Data Extraction

Data were extracted by 1 reviewer, and key data points from the studies, specified in the protocol and identified on further study of the publications, were recorded in a spreadsheet and validated by a second reviewer. The data extraction form was based on the minimum requirements recommended by the Cochrane Handbook for Systematic Reviews [27]. The types of data extracted from the studies are shown in Table 1.

**Table 1:** Data extracted from the studies.

Article information	Data extracted
General study information	Title of publication
	Year of publication
	Authors
Study characteristics	Study design
	Country of study
	Study population
	Analyzed sample size
	Comparators
	Study duration
Characteristics of the conversational agents	Name of conversational agents
	Architecture
	Device or platform on which agent is accessed
	Intended user
	Primary purpose
Intended outcomes of the conversational agents	Health objective (general)
	Health objective (specific)
Evaluation	Effectiveness in achieving intended purpose
	Health literacy
	Improvement in health care provision
	Health care resource implications
	Usability
	Acceptability or satisfaction
	User perceptions qualitative feedback
	Conclusions
	Implications for future study

### Risk-of-Bias and Quality Assessment

All quality assessments were conducted by 2 independent reviewers, with disagreements resolved by consensus. If this was not possible, the opinion of a third reviewer was sought. As there was a wide variety of study designs, the study types were classified by 1 reviewer and validated by a second reviewer, with disagreements being resolved by discussion with a third reviewer. As the broad inclusion criteria were intended to capture all relevant studies, a few of the included studies used implementation models for artificial AI research that were beyond the scope of classic public health design methods. This resulted in some study designs being categorized as *other*.

The Cochrane Collaboration risk-of-bias tool was used to evaluate the risk of bias in randomized controlled trials (RCTs) [28]. The CASP (Critical Appraisal Skills Programme) tools for cohort and qualitative studies were used for the respective studies [29], and the Appraisal tool for Cross-Sectional Studies (AXIS) tool was used to assess the quality of cross-sectional survey studies [30]. Studies that were coded as *other* design types were also assessed using the AXIS tool, which was deemed to be the most rigorous and appropriate tool because it systematically evaluates elements of the introduction, methods, results, and discussion sections, and is not limited to the RCT-specific questions used in the risk-of-bias tool.

The results of the Cochrane Collaboration risk-of-bias tool were summarized using RevMan 5.3. CASP and AXIS scores were calculated using yes=1, no=0, and cannot tell or do not know=0 for each question. The scores for each question were summed to provide a score for each study, which were averaged according to study type and are presented in the results.

### Data Analysis and Synthesis

Due to the variability in populations, interventions, outcomes*,* and study designs, a meta-analysis of the studies was not possible. Therefore, we report a structured analysis of the findings to draw conclusions about the effectiveness and user perceptions of conversational agents in health care. For the purpose of this review, the agent was considered effective if there was a statistically significant (*P*<.05) improvement in a given outcome as compared with a comparator or control, or over time. If no significance was reported or the difference was nonsignificant or significantly worse between groups or over time, the agent was considered to have no significant evidence supporting it. Limitations and future directions for research were also summarized.

The synthesis framework for the assessment of health information technology (SF/HIT) was used to structure the evaluation of the studies because it included a whole system set of outcome variables [31]. These included effectiveness, satisfaction, and perceived ease of use or usefulness, among others. In accordance with the framework, evidence for each of the outcome variables was coded as *positive or mixed* or *neutral or negative*. If the study did not address the outcome in question, it was coded as *neutral or negative*.

Finally, where qualitative user feedback was reported by the studies, it was examined to extract common themes by extracting the sections of the original text that discussed the qualitative perceptions, reducing them to key themes, and then comparing those key themes across the different studies.

## Results

### Included Studies

**Figure 1:**
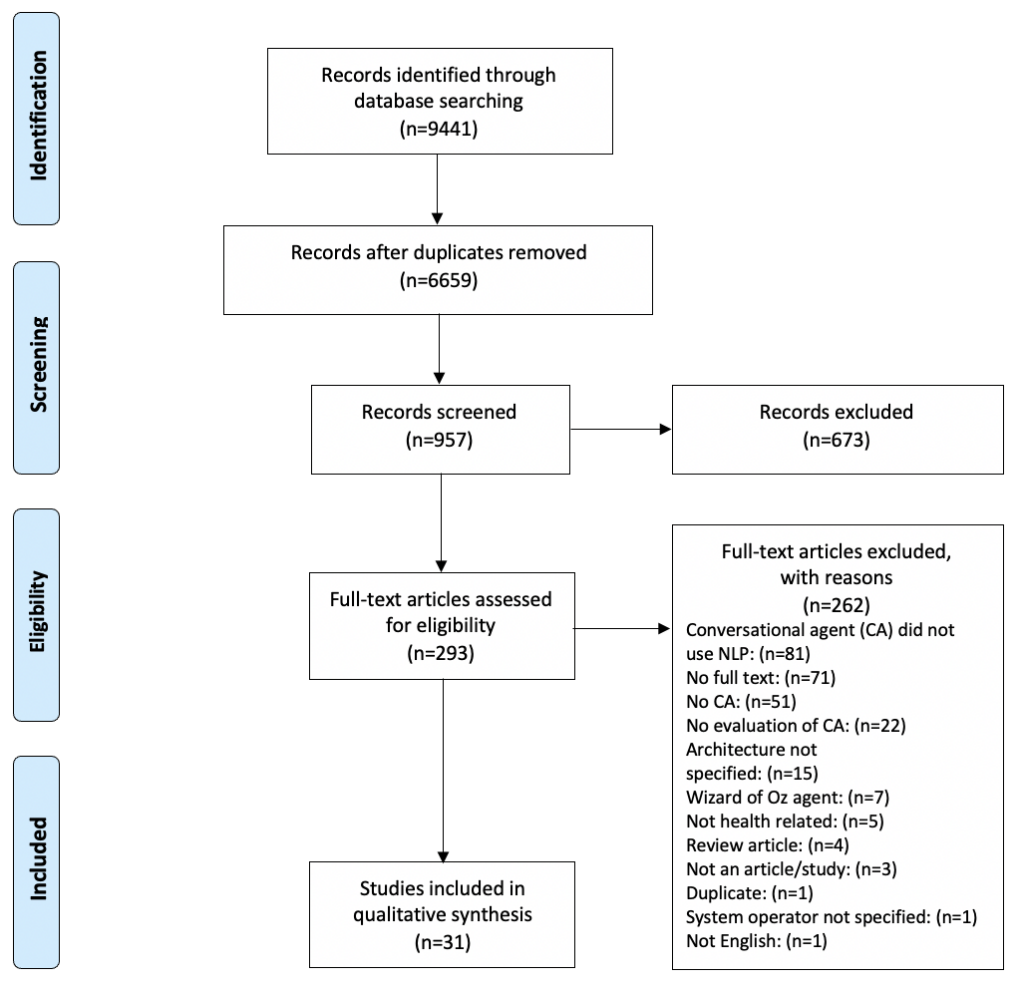
Preferred Reporting Items for Systematic Review and Meta-Analyses flow diagram. NLP: natural language processing.

Overall, 9441 studies were retrieved from the 6 databases, of which 2782 were duplicates. The reference management software EndNote was used for initial screening, with keywords based on the original search categories used to exclude studies that did not meet the criteria. After 6 passes, 957 citations remained for abstract screening. The primary reason for exclusion at the screening stage was that the study did not include an interactive, responsive conversational agent (n=470), was a review paper (n=65), was not health-related (n=48), or did not report any evaluation of the conversational agent (n=46). Of these 957 citations, 293 were selected for full-text review. In the final review, 31 papers were included. The reasons for exclusion after full-text review are detailed in Figure 1, with the most common reason being that the conversational agent did not use NLP (n=81), the full text was not available (n=71), or there was no conversational agent in the study (n=51).

### Study Characteristics

The characteristics of the 31 included studies are summarized in Multimedia Appendix 3 [8,9,12-15,32-56]. Of these studies, 45% (14/31) evaluated conversational agents that had some type of audio or speech element. Of the agents, 45% (14/31) were chatbots (including 2 voice chatbots and 1 chatbot that also used a wizard), 19% (6/31) were ECAs (including 1 virtual doctor), and 10% (3/31) were interactive voice response (IVR) phone calls, virtual patients, and speech recognition screening systems. The final 2 comprised a contextual question-answering agent and a voice recognition triage system. In the 26 studies that reported the device that their conversational agent was used on; 35% (9/26) used computers, 27% (7/26) used web-based apps, 23% (6/26) used mobile phone apps, 15% (4/26) used telephone calls; 1 study used a tablet (the percentages do not add up to 100% because one agent could be used on a computer and also the telephone).

There were a wide variety of areas of health care targeted by the conversational agents of the included studies. The largest proportion of them (12/31, 39%) addressed mental health issues [13,32-42], with 19% (6/31) providing some form of clinical decision or triage support [8,12,40,42-44] and treatment support (including encouraging users to get screened) [9,45-49], 10% (3/31) being used to support training of health care students [15,41,50] and the screening or diagnosis of users [14,38,51], 7% (2/31) targeting physical health [52,53] and layperson medical education [54,55]; 1 agent was designed to help monitor users’ speech [56]. The percentages do not add up to 100% because some of the studies that addressed mental health also fit into one of the other categories.

The study designs also varied widely, with 29% (9/31) using cross-sectional designs, 26% (8/31) using RCTs, 23% (7/31) using qualitative methods, 19% (6/31) using cohort studies, and 1 using a cluster crossover design. The full data extraction table is available in Multimedia Appendix 4 [8,9,12-15,32-56].

### Overall Evaluation of Conversational Agents

Overall, about three-quarters of the studies (22/30, 73%) reported positive or mixed results for most of the outcomes. A total of 8 studies were coded as reporting positive or mixed evidence for 10 or more of the 11 outcomes specified in the SF/HIT; the analysis for this review was limited to the interpretation of impact as reported by study authors to reflect evaluation outcomes. Excluding 1 study, which was an acceptability study only and did not assess the other outcomes, the average number of outcomes that were coded as *positive or mixed* was 67% (7.4/11, SD 2.5). However, the number of outcomes met per study ranged from 1/11 to 11/11 (9-100%). Perceived ease of use or usefulness (27/30, 90%), the process of service delivery or performance (26/30, 87%), appropriateness (24/30, 80%), and satisfaction (26/31, 84%) were the outcomes that had the most support from the studies. Just over three-quarters (23/30, 77%) of the studies also reported positive or mixed evidence of effectiveness.

However, very few studies discussed the cost-effectiveness (5/30, 17%, coded as *positive or mixed*) or safety, privacy, and security (14/30, 47%, coded as *positive or mixed*) outcomes for the agents being evaluated. About a quarter of studies (8/30, 27%) had neither positive nor mixed reported evidence for more than half of the SF/HIT outcomes. The evaluation of the SF/HIT outcomes is summarized in Table 2 [31].

**Table 2:** Summary of the studies based on the evaluation outcomes from the synthesis framework for the assessment of health information technology^a^.

First author (reference)	Preventive care	Adherence or attendance	Efficiency	Perceived ease of use or usefulness	Effectiveness	Performance	Safety or privacy or security	Acceptability	Cost-effectiveness	Appropriateness	Satisfaction	n (%)
Adams [9]	1	1	1	1	1	1	1	1	0	1	1	10 (91)
Bibault [46]	1	1	1	1	1	1	1	1	0	1	1	10 (91)
Borja-Harta [50]	0	1	1	1	1	1	1	0	0	1	0	7 (64)
Cameron [32]	0	0	1	1	0	1	0	1	0	0	1	5 (45)
Chaix [45]	1	0	1	1	1	1	1	0	0	1	1	8 (73)
Chang [8]	0	1	0	1	1	0	1	1	0	1	1	7 (64)
Crutzen [54]	0	1	1	1	1	1	1	1	0	1	1	9 (82)
Dimeff [42]	1	0	1	1	1	1	1	1	1	1	1	10 (91)
Elmasri [33]	0	0	0	1	0	1	1	0	0	1	1	5 (45)
Fitzpatrick [13]	1	1	1	1	1	1	1	1	0	1	1	10 (91)
Friederichs [53]	0	0	0	1	0	1	0	1	0	0	1	4 (36)
Fulmer [34]	1	1	0	0	1	1	1	0	0	0	1	6 (55)
Galescu [52]	0	0	1	1	0	1	0	0	0	0	0	3 (27)
Ghosh [44]	1	1	1	1	1	1	0	1	0	1	1	9 (82)
Havik [14]	1	1	1	1	1	1	0	1	1	1	1	10 (91)
Heyworth [47]	0	1	1	1	1	1	1	1	0	1	0	8 (73)
Hudlicka [35]	1	1	1	1	1	1	1	1	1	1	1	11 (100)
Inkster [36]	1	1	1	1	1	1	0	1	0	1	1	9 (82)
Ireland [56]											1	1 (100)
Isaza- Restrepo [15]	1	1	1	1	1	1	0	1	1	1	1	10 (91)
Ly [37]	0	1	0	1	0	1	0	0	0	1	1	5 (45)
Nakagawa [12]	1	0	1	1	1	1	0	0	0	1	1	7 (64)
Philip (2014) [51]	1	1	1	1	1	1	1	1	0	1	1	10 (91)
Philip (2017) [38]	1	1	1	1	1	1	0	1	0	1	1	9 (82)
Rhee [48]	1	1	1	1	1	1	0	1	0	1	1	9 (82)
Simon [49]	0	1	0	1	0	1	1	1	0	1	1	7 (64)
Spänig [43]	0	0	1	0	1	1	0	1	0	1	1	6 (55)
Washburn [41]	1	0	0	1	1	1	0	0	1	0	0	5 (45)
Wong [55]	0	0	0	1	0	0	0	0	0	0	0	1 (9)
Xu [40]	1	0	1	0	1	0	0	0	0	1	1	5 (45)
Yasavur [39]	0	1	1	1	1	0	0	1	0	1	1	7 (64)
n (%)	17 (57)	19 (63)	22 (73)	27 (90)	23 (77)	26 (87)	14 (47)	20 (67)	5 (17)	24 (80)	26 (84)	

^a^Positive or mixed results have been coded as 1, and neutral or negative results as 0.

When grouped by the agent’s health care scope, studies of certain types of agents appear to do better than others (Table 3). Studies examining screening or diagnosis agents and treatment support agents had the highest average number of positive or mixed outcomes (mean 10, SD 0.6, and mean 9, SD 1.2, respectively). Treatment support agents had primary functions that included empowering patients to engage more fully in clinical appointments, encouraging attending screenings for health care conditions, and supporting patient self-management. In contrast, mental health agents focused on addressing challenges related to depression, anxiety, and alcohol abuse, among others. However, given the small number of studies for each category of agents, these comparisons should be interpreted with caution.

**Table 3:** Summary of evaluation outcomes by the area of health care addressed by the conversational agent^a^.

Agent focus	Number of studies	Average number of outcomes coded *positive or mixed*, n (%)	Range of scores (SD)
Mental health [13,32-42]	12	7 (66)	5-11 (2.4)
Clinical decision or triage support [8,12,40,42-44]	6	7 (67)	5-10 (1.9)
Treatment support [9,45-49]	6	9 (79)	7-10 (1.2)
Health care training (students) [15,41,50]	3	7 (67)	5-10 (2.5)
Screening or diagnosis [14,38,51]	3	10 (88)	9-10 (0.6)
Health care education (laypeople) [54,55]	2	5 (45)	1-9 (5.7)
Physical health [52,53]	2	4 (32)	3-4 (0.7)

^a^The number of studies does not add up to 31 because some studies fit into 2 categories, and the study on monitoring speech was not included because it only addressed 1 of the 11 outcomes. The percentages associated with the average number of outcomes varied slightly because of rounding.

### Qualitative User Perceptions

A total of 18 of the 31 studies included more specific user feedback. The most frequently raised issue with conversational agents (9 studies) was poor understanding because of limited vocabulary, voice recognition accuracy, or error management of word inputs [13,32-37,41,52]. Related to this issue, as the conversational agents often had to ask questions more than once to be able to process the response, users in 3 studies noted disliking the repetitive conversations with the agents [13,36,37]. Both of these issues are key areas of improvement for future research and development of conversational agents because they represent limitations in the usability of the agents in a real-world context.

Feedback from users in 5 studies expressed a preference for interactivity, with users in 1 study noting that they liked the interactivity of the chatbot [35,37], and users in the other 4 studies expressed a desire for greater interactivity or relational skills in the conversational agent [14,32,34,53]. Similarly, users in 4 studies reported liking that the agent had a personality and/or showed empathy [13,32,34,42], whereas users in other studies reported disliking the lack of personal connection or had difficulty in empathizing with the agent [35,37,50] or reported disliking its limited conversation and responses [35,56].

Due to the wide variety of conversational agents, their aims and health care contexts, much of the qualitative user perception data concerned distinct aspects of the agents. However, several studies reported feedback concerned with customization or availability of feature options, with 2 studies commenting on it positively (eg, having both voice and touch modes to allow hands-free work and rapid data input on a triage system for nurses) [8,35], and 3 studies desiring more features and more control [33,37,48]. Additionally, users in 2 studies suggested that better integration of the agent with electronic health record (EHR) systems (for a virtual doctor [42]) or health care providers (for an asthma self-management chatbot [48]) would be useful.

Other features of the agents that users reported liking were the reminders and assistance in forming routines [37,48] and that the agents provided accountability [13,34,48], facilitated learning [13,34,37], and were easy to learn and use [8,15]. In the included studies, 3 of the conversational agents were virtual patients, and users in all 3 studies reported liking that it provided a platform for risk-free learning because they were not practicing on real patients [15,41,50].

Several studies reported user feedback that was specific to that conversational agent. This included a preference for telephone IVR over web-based pediatric care guidance [9] and a simple avatar with a computer-generated voice over a more life-like agent with a recorded voice [42]. Users in 1 study reported liking that the agent initiated conversations [37]. There was opposite feedback in 2 studies about the format of the response, with users preferring preformatted options for one chatbot [36], whereas some users preferred the free-text responses for a diagnostic chatbot because it allowed them to provide contextual information. In contrast, others found it more difficult to know how to respond so the agent would understand [14].

Other agent-specific negative feedback was that the virtual doctor did not have the ability to go deep enough or provide access to other materials [42], that too much information was provided [13,33] or the interaction was too long [13], the use of nonverbal expressions by the avatar [35], and a lack of clarity regarding the aim of the chatbot [37]. Some students who used the virtual patients also reported that it was difficult to empathize [50] and that the agent did not sufficiently encompass real situational complexity [15]. The variety of specific feedback reports demonstrates the importance of examining usability for individual conversational agents and tailoring the design to the intended population. Although there were some preferences and complaints that were frequently reported, much of the feedback was agent dependent. A summary of the thematic analysis is included in Multimedia Appendix 5.

### Implications for Health Care Provision and Resources

Unfortunately, only a few of the studies discussed any improvement in health care provision or implications for resources; 2 of the studies that suggested improvement in health care provision were evaluating virtual patients [41,50], and students in 1 study reported significantly increased confidence in their clinical skills and ability to interview patients. Over 80% of users also reported that the agents helped them follow their treatment more effectively [45] and be more prepared for pediatric visits [9]. In a study of an ECA for sleep disorder screening, 65% of users reported thinking that the agent could provide significant assistance to physicians [51]. Regarding resource implications, the study of a preparatory IVR phone call before pediatric visits found that visit time was significantly reduced in the IVR group compared with the control group [9]. The use of an ECA to screen for depression [38] and a virtual doctor for suicidal patients in emergency departments (EDs) [42] were suggested by the authors to save physicians’ time and reduce the costs associated with ED visits for suicidal ideation, but these outcomes were not evaluated. Similarly, another study suggested that mindfulness meditation could be of more use with more cost-effective training made available via a virtual coach [35].

Suggestions such as this, that conversational agents have the potential to improve health care provision, save health care providers’ time, and reduce costs, were frequently made in the studies. However, as demonstrated above, very few studies quantified these claims and even fewer measured these outcomes with objective measures. This is a limitation of the studies as a whole. Although many were in the early stages of testing, claims about the potential value to the health care system in terms of time or money should be substantiated. However, as evidenced by the number of *neutral or negative* coding in the evaluation, many of the studies did not consider whole system implementation outcomes. It will be important for the future development of conversational agents to consider outcomes such as these from the beginning so that agents that are not only acceptable and usable but also provide value to the health care system can be built.

### Risk-of-Bias and Quality Assessments

There were a variety of study types included in this review; so several different quality assessment tools were used to assess the risk of bias and quality of the 31 included studies. A total of 6 studies could not be classified as RCTs, cohort, qualitative, or cross-sectional studies, and their study design was coded as *other* [12,39,40,44,52,55]. Most of these studies were papers describing the development and initial evaluation of conversational agents, and half of them did not have participants [40,44,55]. Initially, studies that did not have an explicit design were classified as qualitative or interpretative studies. However, on further analysis, many of the studies did not fit the criteria for qualitative studies - evaluating subjective, thematic, and non-numerical data - because they evaluated performance metrics such as word error rates [52], accuracy [12,39,40,52,55], precision [44], and user experience quantified on Likert scales [39]. Therefore, these studies were coded as *other* and assessed using the AXIS tool for cross-sectional studies, which was deemed to provide the most systematic evaluation of the various elements of the studies [30]. The quality of these studies was assessed as best as possible; however, the judgments should be considered in the context of these limitations.

**Figure 2:**
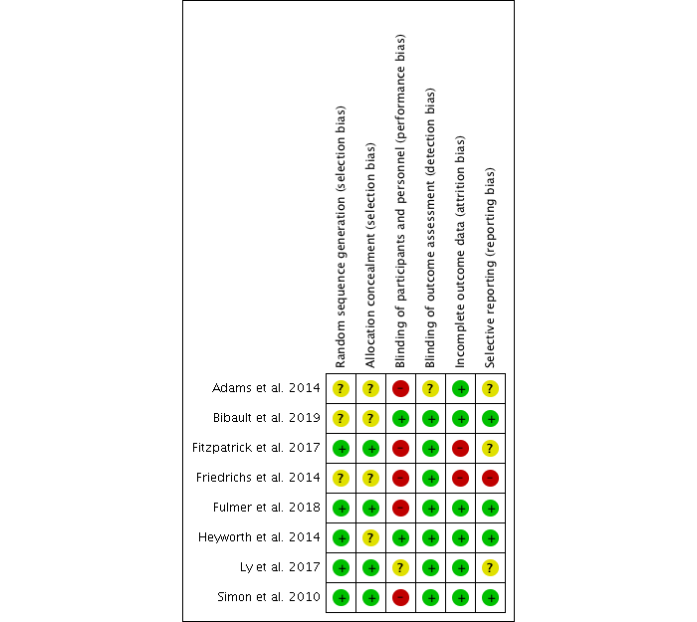
Risk of bias summary: review authors' judgements about each risk of bias item for each included study.

**Figure 3:**
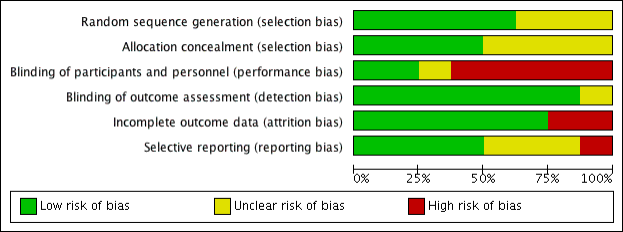
Risk of bias graph: review authors' judgements about each risk of bias item presented as percentages across all included studies.

Overall, the quality of the studies was poor to moderate. On average, RCTs [9,13,34,37,46,47,49,53] and qualitative studies [41,48,56] evaluated were generally determined to have the highest quality and lowest risk of bias, with none of the other 3 study types meeting more than half the criteria for quality assessment. The evaluation of the risk of bias for the 8 RCTs (Figure 2) was carried out using the Cochrane Collaboration risk-of-bias tool [28], and the results were summarized using RevMan 5.3 software (Cochrane) [57]. Overall, the RCTs performed fairly well in the risk-of-bias assessment (Figure 3). About half the studies were assessed as having a low risk of selection bias because of proper random sequence generation (5/8) and allocation concealment (4/8), and a low risk of reporting bias (4/8), as outcomes reported could be compared with a priori protocols or trial registrations. Most studies reported blinding of outcome assessors (7/8) and a low risk of attrition bias because of low or equal dropout across groups or the use of intention-to-treat analyses (6/8). Most of the studies (5/8) had a high risk of performance bias, but this was predominantly because blinding was not possible given the nature of the intervention.

The cohort (n=9) and qualitative (n=3) studies assessed using the CASP checklists met, on average, 5/12 (range 1-10) and 7/10 (range 4-9) criteria, respectively [29]. Of the cohort studies, the questions with the best performance were, “Did the study address a clearly focused issue?” (8/9 yes), “Was the follow up long enough?” (8/9 yes), and “Do the results of this study fit with other available evidence?” (6/9 yes). Studies performed the worst, either by failing to meet the criteria or failing to report it, on questions about cohort recruitment (1/9 yes), identifying and accounting for confounding factors (1/9 yes), accurate exposure and outcome measurement (2/9 and 3/9 yes, respectively), and the applicability of results to the local population (3/9 yes). The qualitative studies, on the other hand, performed best on the questions about whether the qualitative methodology was appropriate, the consideration of ethical issues, clear statements of findings, and whether the results would help locally (3/3 yes for each). None of the 3 studies reported any consideration of the relationship between researcher and participant. They also performed poorly on questions about sample recruitment, data collection, and data analysis (1/3 yes for each).

The cross-sectional (n=5) and *other* (n=6) studies assessed using the AXIS tool met, on average, 50% (range 26-80%) and 42% (range 29-70%) of the criteria, respectively [30]. Percentages are reported instead of the exact number of criteria because several of the questions were not applicable to the studies; so the total number of criteria assessed per study was not the same (averages 19 and 16; ranges 18-20, and 10-19, respectively). Overall, the cross-sectional studies performed best on questions about the clarity of aims (5/5 yes), appropriate outcome variables for the aims (5/5 yes), internal consistency (5/5 yes), and adequate description of basic data (4/5 yes). They performed worst on questions about sample selection—whether it was taken from an appropriate base to represent the population (1/5 yes) and whether the process was likely to select a representative sample (0/5 yes)—the use of appropriate outcome measures (previously assessed; 0/5 yes), whether the methods were adequately described for replication (1/5 yes), and conflicts of interest (1/5 no, most did not report).

The *other* studies performed best on the questions about whether the study design was appropriate for the aims and whether the conclusions were justified by the results (6/6 yes for both). They also did well, overall, on the appropriate choice of outcome variables and internal consistency (5/6 yes for both). However, all the *other* studies for which the questions were applicable performed poorly on questions about the justification of sample size (0/5 yes), whether the selection process was likely to get a representative sample (0/5 yes), addressing nonresponders (0/2 yes), adequate description of basic data (0/4 yes), concerns about nonresponse bias (0/3 no), the presentation of results for all the analyses described in the methods (0/6 yes, although this was mostly because analyses were not adequately described in the methods), and conflicts of interest (0/6 no, again because nothing was reported). Furthermore, only 1 study adequately addressed the questions about the use of previously assessed outcome measures (1/5 yes), sufficient description of the methods for replication (1/6 yes), and discussion of study limitations (1/6 yes). It should be noted that the AXIS tool used to assess the *other* studies was designed for cross-sectional studies and does not fit exactly with the designs of these studies. Therefore, it is possible that these studies would perform better when assessed by a tool specific to their study type. Tables depicting the judgments for each question of the CASP cohort and qualitative checklists and the AXIS tool for the cross-sectional and *other* studies are included in Multimedia Appendices 6-9 [8,12,14,15,32,33,35,36,38-45,48,50-52,54-56].

## Discussion

### Principal Findings

In this systematic review, we examined 31 studies that evaluated the effectiveness and usability of conversational agents in health care. Overall, studies reported a moderate amount of evidence supporting the effectiveness, usability, and positive user perceptions of the agents. On average, two-thirds of the studies (67%) reported positive or mixed evidence for each evaluation outcome. However, this ranged significantly, with usability, agent performance, and satisfaction having the most support across the studies, and cost-effectiveness receiving hardly any support. It should also be noted that the definitions of *effectiveness* were highly varied and, as evidenced by the methodological limitations identified in the quality assessment, rarely evaluated with the scrutiny expected for medical devices. Although the results reported are promising for the use of conversational agents in health care, there are a number of limitations in both the studies analyzed and the structure of this review that questions the validity of this finding.

With regard to qualitative user perceptions of the agents, specific feedback was very mixed. Users highlighted many positive factors of the agents, particularly their personality and ability to provide empathy and emotional support, that they support learning, they are easy to use and access, and they help them be accountable, all of which support the generally positive evaluations of usability and satisfaction outcomes. However, there were a number of limitations of the agents that were consistently raised across the studies that reported qualitative feedback. These included the following: the agents had difficulty understanding them, the agents were repetitive and not sufficiently interactive, and the users had difficulty forming personal connections with the agents. This suggests that despite the generally positive usability reported by the studies, there are a number of barriers to the successful use of conversational agents in health care that will need to be addressed before they can achieve the greatest impact. It should be noted that this review only included studies of conversational agents that used NLP and that free-text inputs are likely to present greater difficulties for comprehension.

The results of this systematic review are largely consistent with the literature, particularly the previous systematic review evaluating conversational agents in health care [2]. They also found a limited quality of design and evidence in the included studies, with inconsistent reporting of study methods (including methods of selection, attrition, and a lack of validated outcome measures) and conflicts of interest [2]. The previous systematic review identified that high-quality evidence of effectiveness and patient safety was limited, which was also observed in this review. Similarly, it noted that high overall satisfaction was generally reported by the studies, but that the most common issues with conversational agents related to language understanding or poor dialogue management, which is consistent with our findings [2]. Some of this similarity in results is likely because of the overlap in included studies; 7 of their 17 included studies were also included in our review [2].

### Quality of the Evidence

As noted in a previous systematic review [2], there were significant issues with the quality of many of the included studies. One of the consistent issues among many of them was a high risk of selection bias. A large proportion of the studies relied on volunteers for the study, many of whom were recruited via self-selection means such as flyers and emails or by downloading the app being studied. The risk with self-selection recruitment is that participants who elect to take part in the study are already more positively predisposed to new technologies than those who do not participate, and would tend to evaluate the technology more positively. To make matters worse, several of the studies also did not sufficiently report their recruitment strategies, and so their potential selection bias cannot be accurately evaluated. In research such as this, where user perceptions are a main outcome, this is a serious concern. Future studies should take care to implement recruitment strategies that minimize this risk of selection bias or balance the potential bias in evaluations by actively recruiting participants who are less inclined toward new technology.

Another limitation of many of the studies was the small sample size. Almost two-thirds of the studies (19/31) used samples of less than 100 participants or items of analysis (eg, voice clips and clinical scenarios) with a median sample size of 48 across all the studies. Many also did not sufficiently report demographic data or whether their sample was representative of their target population. Although many of these studies were early feasibility and usability trials, this is an important issue to address in future research testing these agents to determine whether an agent will be used and used effectively by its target population.

### Limitations

The validity of the evidence extracted from the included studies was also affected by limitations in the structure of this review. The SF/HIT was used to provide a structured set of whole system implementation outcomes to evaluate the conversational agents [31]. However, an issue with the use of this framework, which was discovered during analysis, was that many of the included studies were describing system innovation. Therefore, they did not address or provide evidence for many of the outcomes described by the SF/HIT. Additionally, as the included data indicated a self-reported impact in the studies of effectiveness, the study effectiveness is biased favorably toward the authors’ reporting of impact.

This limitation in the use of the framework for this review also highlights a limitation in many of these studies, namely, that they do not think about whole system implementation from the early stages of agent design, development, and testing. It is possible that the lack of evaluation of the implications of the agents for health care provision and resources was because of an emphasis on technology development and evaluation, rather than system integration. This is a pervasive issue in technological innovation, so much that it drove the development of the nonadoption, abandonment, scale-up, spread, and sustainability framework as a means of predicting and assessing the success of new health technologies [58] and the development and evaluation of new conversational agents to ensure that these later-stage implications of health care provision, cost-effectiveness, and privacy and security are sufficiently considered from the early stages of innovation. They must also be properly evaluated with a large sample of users, rather than be simply presented as unsubstantiated claims that the agent will reduce costs and save health care providers’ time.

Additionally, in accordance with the SF/HIT framework, the impact of outcomes on each outcome was coded as *positive or mixed* or *neutral or negative*. However, this combination of positive and mixed outcomes reduces the granularity of the results. During the coding process, several outcomes were distinctly coded as *positive or mixed*, and collating the 2 outcome impacts into 1 reduces the precision of the information presented to the readers. Additionally, studies that did not assess the outcome in question were coded as *neutral or negative* because they did provide explicit support for the outcome. In the analysis, outcomes were initially coded separately as positive, mixed, positive or mixed (for studies that reported a positive outcome but did not provide sufficient statistical evidence), and neutral or negative. This table is available in Multimedia Appendix 10. Positive and mixed outcomes were combined for the final presentation of the data in line with the framework. However, it might be more useful to distinguish between studies that attempted to find significant evidence for an outcome but did not and those that did not attempt it. This would provide a clearer picture of which outcomes are not being supported by the evidence and should be targeted for improvement, and which outcomes still need to be examined. In the future, it would be worth evaluating whether the coding system should be adjusted to provide a more detailed and informative summary of the evidence.

Further limitations of this review are that we limited the focus to include only unconstrained NLP and interaction. This was chosen as a focus because of the advantages NLP offers for simulating human-to-human interaction. However, it may have excluded studies of relevant conversational agents that could be satisfactory, useful, and effective in addressing current health care challenges. Additionally, no spidering searches were used to identify potentially relevant studies in the references of the included studies that were missed in the initial search. The exclusion of conference abstracts might also have caused relevant papers that were classified as abstracts to be missed; however, a previous systematic review that included conference abstracts in their search only had 1 included in their final selection [2]. The inclusion of only studies published in English is also likely to exclude relevant research on conversational agents conducted in other countries. These limitations should be addressed in future studies to ensure that the full body of relevant literature is examined.

### Future Directions

Future reviews of conversational agents in health care could be extended to include constrained NLP and non-NLP conversational agents. A synthesis of the evidence identified here with other types of conversational agents in health care, perhaps structured according to the taxonomy suggested by Montenegro et al [5], could be used to examine overall trends and provide a better picture of what is being used, what works, and what does not, to further guide the development of conversational agents that are most likely to be successful.

Future research should also include more qualitative evaluations of the features that users like and dislike. Only half (18/31) of the studies included in this review reported specific user feedback, despite the fact that 7 of the remaining 13 studies included some measure of usability or user perceptions. It will be important to identify all of the structural, physical, and psychological barriers to use if conversational agents are to achieve their potential for improving health care provision and reducing the strain on health care resources. To this end, it would be useful for future studies to structure their evaluation of conversational agents around a behavioral change framework (eg, the Behavior Change Wheel framework [59]). This is important not only when evaluating the effectiveness of behavior change-focused conversational agents, but also when determining whether and how the adoption of new conversational agent technology will be successful.

It will be important for future studies of conversational agents to take care to properly structure and report their studies to improve the quality of the evidence. Without high-quality evidence, it is difficult to assess the current state of conversational agents in health care - what is working, and what needs to be improved to make them a more useful tool. Similarly, there is a gap in the evidence regarding the health economics of these agents. Very few studies in this review even discussed the cost analysis of the agent in questions, let alone provide substantive evidence about its cost-effectiveness. The evaluation of costs and outcomes of new technologies and their privacy, security, and interoperability will be necessary to advance value-based health care [60]. However, there is very little evidence to suggest that the conversational agents examined in this review considered or addressed these concerns. User feedback on 2 of the studies even noted that better interoperability between the agent and EHRs or health care providers would improve its usefulness.

### Conclusions

The objective of this systematic review was to synthesize evidence of conversational agents’ usability, effectiveness, and satisfaction in health care. Although the studies generally reported positive outcomes relating to the agents’ usability and effectiveness, the quality of the evidence was not sufficient to provide strong evidence to support these claims. This study extended the literature by expanding its summary to examine a whole system set of evaluation outcomes, including cost-effectiveness, privacy, and security, which have not been systematically examined in previous reviews. In addition, it provides a distinct contribution by conducting a thematic analysis of the qualitative user perceptions of the agents. Further research is needed to examine the cost-effectiveness and value of these agents in health care, both in their current and potential states. Higher-quality studies—with more consistent reporting of design methods and better sample selection—are also needed to more accurately assess the usefulness and identify the key areas of improvement for current conversational agents. A more holistic approach to the design, development, and evaluation of conversational agents will help drive innovation and improve their value in health care.

## Appendix

Multimedia Appendix 1 Search queries and number of results for each database.

Multimedia Appendix 2 EndNote search details.

Multimedia Appendix 3 Summary of study characteristics.

Multimedia Appendix 4 Data extraction table.

Multimedia Appendix 5 Summary of the thematic analysis of qualitative user feedback.

Multimedia Appendix 6 Summary of the quality assessment and judgments of cohort studies using the CASP (Critical Appraisal Skills Programme) Cohort Study Checklist.

Multimedia Appendix 7 Summary of the quality assessment and judgments of qualitative studies using the CASP (Critical Appraisal Skills Programme) Qualitative Study Checklist.

Multimedia Appendix 8 Summary of the quality assessment and judgments of the cross-sectional studies using the Appraisal tool for Cross-Sectional Studies tool.

Multimedia Appendix 9 Summary of the quality assessment and judgments of the ‘other’ studies using the Appraisal tool for Cross-Sectional Studies tool.

Multimedia Appendix 10 Summary of the studies based on the evaluation outcomes from the synthesis framework for the assessment of health information technology differentiating between positive and mixed outcomes.

Multimedia Appendix 11 PRISMA (Preferred Reporting Items for Systematic Review and Meta-Analyses) checklist.

## Abbreviations

AI: artificial intelligence
AXIS: Appraisal tool for Cross-Sectional Studies
CASP: Critical Appraisal Skills Programme
ECA: embodied conversational agent
ED: emergency department
EHR: electronic health record
IVR: interactive voice response
NLP: natural language processing
PRISMA: Preferred Reporting Items for Systematic Review and Meta-Analyses
RCT: randomized controlled trial
SF/HIT: synthesis framework for the assessment of health information technology
