# Supplementary material for: The Effectiveness of Artificial Intelligence Conversational Agents in Health Care: Systematic Review
Source: J Med Internet Res. 2020 Oct 22;22(10):e20346. doi: 10.2196/20346 (PMC7644372; doi:10.2196/20346)
Supplement: Multimedia Appendix 1 [file jmir_v22i10e20346_app1.docx]

### Multimedia Appendix A. Search queries and number of results for each database

| **Database** | **Search terms** | **Retrieved** |
| --- | --- | --- |
| **PubMed** | ((((Speech recognition software[mh] or "Conversational agent*"[tiab] or "embodied conversational agent*"[tiab] or chatbot*[tiab] or avatar*[tiab] or "dialog* system"[tiab] or "speech recognition software"[tiab] or "voice recognition software"[tiab] or "virtual assistan*"[tiab] or "virtual nurs*"[tiab] or "virtual patient"[tiab] or "virtual coach*"[tiab] or "virtual agent"[tiab] or "relation* agent"[tiab] or "assistance technol*"[tiab] or "intelligent assistan*"[tiab] or "digital assistan*"[tiab] or "natural language interface"[tiab] or "interactive computer agent"[tiab] or "computer-assisted instruction"[tiab] or "natural language communication"[tiab] or "natural language understanding"[tiab] or "unconstrained natural language processing"[tiab])) AND (Health facilities[mh] or Health communication[mh] or Health services[mh] or exp health services accessibility[mh] or Delivery of healthcare[mh] or exp Health behavior[mh] or Exercise[mh] or Simulation training[mh] or Health education[mh] or health literacy[mh] or "patient acceptance of healthcare"[mh] or health knowledge, attitudes, practice[mh] or "treatment adherence or compliance"[mh] or asthma[mh] or sex education[mh] or exp aged[mh] or exp counseling[mh] or smoking cessation[mh] or exp diet[mh] or exp education, medical[mh] or exp substance-related disorders[mh] or social skills[mh] or autism spectrum disorder[mh] or patient education as topic[mh] or diabetes mellitus[mh] or cardiovascular disease[mh] or pulmonary disease, chronic obstructive[mh] or "healthcare delivery"[tiab] or "healthcare access"[tiab] or health[tiab] or healthcare[tiab] or behavio?r[tiab] or exerci*[tiab] or diet[tiab] or "simulation training"[tiab] or education[tiab] or "elderly care"[tiab] or "sex* education"[tiab] or "health literacy"[tiab] or "counsel?ing"[tiab] or "well-being"[tiab] or "smoking cessation"[tiab] or "cognitive dysfunction"[tiab] or "mental health"[tiab] or "social skills"[tiab] or "autism spectrum disorder"[tiab] or diabetes[tiab] or "heart health"[tiab] or "chronic obstructive pulmonary disease"[tiab] or "COPD"[tiab] or "sun protection"[tiab] or "physical activity"[tiab]))) AND ("Outcome Assessment (Health Care)"[mh] or program evaluation[mh] or feasibility studies[mh] or pilot projects[mh] or "diffusion of innovation"[mh] or cost-benefit analysis[mh] or "Reproducibility of results"[mh] or Feasib*[tiab] or usab*[tiab] or evaluat*[tiab] or outcome*[tiab] or acceptability[tiab] or acceptance[tiab] or "treatment adherence"[tiab] or effectiv*[tiab] or adoption[tiab] or assess*[tiab] or "user experience*"[tiab] or efficacy[tiab] or utility[tiab] or utili?ation[tiab] or "patient* acceptance"[tiab] or "patient* acceptability"[tiab] or "user* acceptance"[tiab] or "user* acceptability"[tiab] or "user* perce*"[tiab] or "patient* perce*"[tiab] or "user* perspective*"[tiab] or "patient* perspective*"[tiab] or "user* view*"[tiab] or "patient* view*"[tiab] or cost*[tiab]) | 1065 |
| **Medline (OVID)** | (Speech recognition software/ or ((Conversational adj1 agent*) or (embodied adj2 agent*) or chatbot* or avatar* or (dialog* adj1 system) or speech recognition software or voice recognition software or (virtual adj1 (assistan* or nurs* or patient or coach* or agent)) or (relation* adj1 agent) or assistance technol* or (intelligent adj2 assistan*) or (digital adj2 assistan*) or natural language interface or interactive computer agent or computer-assisted instruction or natural language communication or natural language understanding or unconstrained natural language processing).ti,ab.) AND (Health facilities/ or Health communication/ or Health services/ or exp health services accessibility/ or Delivery of healthcare/ or exp Health behavior/ or Exercise/ or Simulation training/ or Health education/ or health literacy/ or "patient acceptance of healthcare"/ or health knowledge, attitudes, practice/ or "treatment adherence or compliance"/ or asthma/ or sex education/ or exp aged/ or exp counseling/ or smoking cessation/ or exp diet/ or exp education, medical/ or exp substance-related disorders/ or social skills/ or autism spectrum disorder/ or patient education as topic/ or diabetes mellitus/ or cardiovascular disease/ or pulmonary disease, chronic obstructive/ or (healthcare delivery or healthcare access or health or healthcare or behavio?r or exerci* or diet or simulation training or education or elderly care or sex* education or health literacy or counsel?ing or well-being or smoking cessation or cognitive dysfunction or mental health or social skills or autism spectrum disorder or diabetes or heart health or chronic obstructive pulmonary disease or COPD or sun protection or physical activity).ti,ab.) AND ("Outcome Assessment (Health Care)"/ or program evaluation/ or feasibility studies/ or pilot projects/ or "diffusion of innovation"/ or cost-benefit analysis/ or "Reproducibility of results"/ or (Feasib* or usab* or evaluat* or outcome* or acceptability or acceptance or treatment adherence or effectiv* or adoption or assess* or user experience* or efficacy or utility or utili?ation or patient* acceptance or patient* acceptability or user* acceptance or user* acceptability or user* perce* or patient* perce* or user* perspective* or patient* perspective* or user* view* or patient* view* or cost*).ti,ab.) | 1599 |
| **Embase (OVID)** | (Automatic speech recognition/ or ((Conversational adj1 agent*) or (embodied adj2 agent*) or chatbot* or avatar* or (dialog* adj1 system) or (dialog* adj1 agent) or speech recognition software or voice recognition software or (virtual adj1 (assistan* or nurs* or patient or coach* or agent)) or (relation* adj1 agent) or assistance technol* or (intelligent adj2 assistan*) or (digital adj2 assistan*) or natural language interaction or interactive computer agent or computer-assisted instruction or natural language communication or natural language understanding or unconstrained natural language processing).ti,ab.) AND (Health care facility/ or medical information/ or Health service/ or exp healthcare access/ or healthcare delivery/ or exp Health behavior/ or Exercise/ or Simulation training/ or Health education/ or health literacy/ or patient attitude/ or attitude to health/ or patient compliance/ or asthma/ or sexual education/ or exp aged/ or exp counseling/ or smoking cessation/ or exp diet/ or exp medical education/ or exp drug dependence/ or social competence/ or autism/ or patient education/ or diabetes mellitus/ or cardiovascular disease/ or chronic obstructive lung disease/ or (healthcare delivery or healthcare access or health or healthcare or behavio?r or exerci* or diet or simulation training or education or elderly care or sex* education or health literacy or counsel?ing or well-being or smoking cessation or cognitive dysfunction or mental health or social skills or autism spectrum disorder or diabetes or heart health or chronic obstructive pulmonary disease or COPD or sun protection or physical activity).ti,ab.) AND (Outcome assessment/ or program evaluation/ or feasibility study/ or pilot study/ or mass communication/ or cost benefit analysis/ or reproducibility/ or (feasib* or usab* or evaluat* or outcome* or acceptability or acceptance or treatment adherence or effectiv* or adoption or assess* or user experience* or efficacy or utility or utili?ation or patient* acceptance or patient* acceptability or user* acceptance or user* acceptability or user* perce* or patient* perce* or user* perspective* or patient* perspective* or user* view* or patient* view* or cost*).ti,ab.) | 2145 |
| **CINAHL** | ((MH Voice recognition systems) OR TI ((Conversational n1 agent*) or (embodied n2 agent*) or chatbot* or avatar* or (dialog* n1 system) or speech recognition software or voice recognition software or (virtual n1 (assistan* or nurs* or patient or coach* or agent)) or (relation* n1 agent) or assistance technol* or (intelligent n2 assistan*) or (digital n2 assistan*) or natural language interface or interactive computer agent or computer-assisted instruction or natural language communication or natural language understanding or unconstrained natural language processing) OR AB ((Conversational n1 agent*) or (embodied n2 agent*) or chatbot* or avatar* or (dialog* n1 system) or speech recognition software or voice recognition software or (virtual n1 (assistan* or nurs* or patient or coach* or agent)) or (relation* n1 agent) or assistance technol* or (intelligent n2 assistan*) or (digital n2 assistan*) or natural language interface or interactive computer agent or computer-assisted instruction or natural language communication or natural language understanding or unconstrained natural language processing)) AND ((MH “Health facilities”) or (MH “Communication”) or (MH “health services accessibility+”) or (MH “Health behavior”) or (MH “exercise”) or (MH “Computerized clinical simulation testing) or (MH “health education”) or (MH “health literacy”) or (MH “Patient attitudes”) or (MH “Attitude to health”) or (MH “patient compliance”) or (MH “asthma”) or (MH “sex education”) or (MH “Aged+”) or (MH “Counseling+”) or (MH “smoking cessation”) or (MH “diet+”) or (MH “Education, medical+”) or (MH “substance dependence+) or (MH “social skills training”) or (MH “Autistic disorder”) or (MH “patient education”) or (MH "diabetes mellitus") or (MH "cardiovascular diseases") or (MH "Pulmonary Disease, Chronic Obstructive") OR TI (healthcare delivery or healthcare access or health or healthcare or behavio?r or exerci* or diet or simulation training or education or elderly care or sex* education or health literacy or counsel?ing or well-being or smoking cessation or cognitive dysfunction or mental health or social skills or autism spectrum disorder or diabetes or heart health or chronic obstructive pulmonary disease or COPD or sun protection or physical activity) OR AB (healthcare delivery or healthcare access or health or healthcare or behavio?r or exerci* or diet or simulation training or education or elderly care or sex* education or health literacy or counsel?ing or well-being or smoking cessation or cognitive dysfunction or mental health or social skills or autism spectrum disorder or diabetes or heart health or chronic obstructive pulmonary disease or COPD or sun protection or physical activity)) AND ((MH “Outcome assessment”) or (MH “Program evaluation”) or (MH “pilot studies”) or (MH “Diffusion of innovation”) or (MH “Cost benefit anaylsis”) or (MH “reproducibility of results”) OR TI (feasib* or usab* or evaluat* or outcome* or acceptability or acceptance or treatment adherence or effectiv* or adoption or assess* or user experience* or efficacy or utility or utili?ation or patient* acceptance or patient* acceptability or user* acceptance or user* acceptability or user* perce* or patient* perce* or user* perspective* or patient* perspective* or user* view* or patient* view* or cost*) OR AB (feasib* or usab* or evaluat* or outcome* or acceptability or acceptance or treatment adherence or effectiv* or adoption or assess* or user experience* or efficacy or utility or utili?ation or patient* acceptance or patient* acceptability or user* acceptance or user* acceptability or user* perce* or patient* perce* or user* perspective* or patient* perspective* or user* view* or patient* view* or cost*)) | 935 |
| **Web of Science** | ((MH Voice recognition systems) OR TI ((Conversational n1 agent*) or (embodied n2 agent*) or chatbot* or avatar* or (dialog* n1 system) or speech recognition software or voice recognition software or (virtual n1 (assistan* or nurs* or patient or coach* or agent)) or (relation* n1 agent) or assistance technol* or (intelligent n2 assistan*) or (digital n2 assistan*) or natural language interface or interactive computer agent or computer-assisted instruction or natural language communication or natural language understanding or unconstrained natural language processing) OR AB ((Conversational n1 agent*) or (embodied n2 agent*) or chatbot* or avatar* or (dialog* n1 system) or speech recognition software or voice recognition software or (virtual n1 (assistan* or nurs* or patient or coach* or agent)) or (relation* n1 agent) or assistance technol* or (intelligent n2 assistan*) or (digital n2 assistan*) or natural language interface or interactive computer agent or computer-assisted instruction or natural language communication or natural language understanding or unconstrained natural language processing)) AND ((MH “Health facilities”) or (MH “Communication”) or (MH “health services accessibility+”) or (MH “Health behavior”) or (MH “exercise”) or (MH “Computerized clinical simulation testing) or (MH “health education”) or (MH “health literacy”) or (MH “Patient attitudes”) or (MH “Attitude to health”) or (MH “patient compliance”) or (MH “asthma”) or (MH “sex education”) or (MH “Aged+”) or (MH “Counseling+”) or (MH “smoking cessation”) or (MH “diet+”) or (MH “Education, medical+”) or (MH “substance dependence+) or (MH “social skills training”) or (MH “Autistic disorder”) or (MH “patient education”) or (MH "diabetes mellitus") or (MH "cardiovascular diseases") or (MH "Pulmonary Disease, Chronic Obstructive") OR TI (healthcare delivery or healthcare access or health or healthcare or behavio?r or exerci* or diet or simulation training or education or elderly care or sex* education or health literacy or counsel?ing or well-being or smoking cessation or cognitive dysfunction or mental health or social skills or autism spectrum disorder or diabetes or heart health or chronic obstructive pulmonary disease or COPD or sun protection or physical activity) OR AB (healthcare delivery or healthcare access or health or healthcare or behavio?r or exerci* or diet or simulation training or education or elderly care or sex* education or health literacy or counsel?ing or well-being or smoking cessation or cognitive dysfunction or mental health or social skills or autism spectrum disorder or diabetes or heart health or chronic obstructive pulmonary disease or COPD or sun protection or physical activity)) AND ((MH “Outcome assessment”) or (MH “Program evaluation”) or (MH “pilot studies”) or (MH “Diffusion of innovation”) or (MH “Cost benefit anaylsis”) or (MH “reproducibility of results”) OR TI (feasib* or usab* or evaluat* or outcome* or acceptability or acceptance or treatment adherence or effectiv* or adoption or assess* or user experience* or efficacy or utility or utili?ation or patient* acceptance or patient* acceptability or user* acceptance or user* acceptability or user* perce* or patient* perce* or user* perspective* or patient* perspective* or user* view* or patient* view* or cost*) OR AB (feasib* or usab* or evaluat* or outcome* or acceptability or acceptance or treatment adherence or effectiv* or adoption or assess* or user experience* or efficacy or utility or utili?ation or patient* acceptance or patient* acceptability or user* acceptance or user* acceptability or user* perce* or patient* perce* or user* perspective* or patient* perspective* or user* view* or patient* view* or cost*)) | 2954 |
| **ACM digital library** | (recordAbstract:(+Speech +recognition +software) OR recordAbstract:(+conversational +agent) OR recordAbstract:(+embodied +agent) OR recordAbstract:(chatbot*) OR recordAbstract:(avatar*) OR recordAbstract:(+dialog* +system) OR recordAbstract:(+voice +recognition +software) OR recordAbstract:(+virtual +assistan*) OR recordAbstract:(+virtual +nurs*) OR recordAbstract:(+virtual +patient) OR recordAbstract:(+virtual +coach*) OR recordAbstract:(+virtual +agent) OR recordAbstract:(+relation* +agent) OR recordAbstract:(+assistance +technol*) OR recordAbstract:(+intelligent +assistan*) OR recordAbstract:(+digital +assistan*) OR recordAbstract:(+natural +language +interface) OR recordAbstract:(+interactive +computer +agent) OR recordAbstract:(+computer +assisted +instruction) OR recordAbstract:(+natural +language +communication) OR recordAbstract:(+natural +language +understanding) OR recordAbstract:(+unconstrained +natural +language +processing)) AND ((recordAbstract:(+health +facilities) OR recordAbstract:(+health +communication) OR recordAbstract:(+health +services) OR recordAbstract:(+health +access) OR recordAbstract:(+healthcare +delivery) OR recordAbstract:(+health +behavior) OR recordAbstract:(+health +behaviour) OR recordAbstract:(+exerci*) OR recordAbstract:(+simulation +training) OR recordAbstract:(+health +education) OR recordAbstract:(+health +literacy) OR recordAbstract:(+health +knowledge) OR recordAbstract:(+health +practice) OR recordAbstract:(+health +attitudes) OR recordAbstract:(+treatment +compliance) OR recordAbstract:(+treatment +adherence) OR recordAbstract:(+asthma) OR recordAbstract:(+sex* +education) OR recordAbstract:(+elderly +care) OR recordAbstract:(+counseling) OR recordAbstract:(+counselling) OR recordAbstract:(+smoking +cessation) OR recordAbstract:(+diet) OR recordAbstract:(+medical +education) OR recordAbstract:(+substance-related +disorders) OR recordAbstract:(+social +skills) OR recordAbstract:(+autism) OR recordAbstract:(+patient +education) OR recordAbstract:(+health) OR recordAbstract:(+healthcare) OR recordAbstract:(+education) OR recordAbstract:(+health +literacy) OR recordAbstract:(+wellbeing) OR recordAbstract:(+cognitive +dysfunction) OR recordAbstract:(+mental +health) OR recordAbstract: (diabetes) OR recordAbstract: (+cardiovascular +disease) OR recordAbstract: (+sun +protection) OR recordAbstract: (+chronic +obstructive +pulmonary +disease) OR recordAbstract: (COPD) OR recordAbstract: (+physical +activity)) AND ((recordAbstract:(+outcome*) OR recordAbstract:(+program +evaluation) OR recordAbstract:(+feasibility +stud*) OR recordAbstract:(+pilot +stud*) OR recordAbstract:(+cost*) OR recordAbstract:(+reproducibility) OR recordAbstract:(+feasib*) OR recordAbstract:(+usab*) OR recordAbstract:(+evaluat*) OR recordAbstract:(+effectiv*) OR recordAbstract:(+adoption) OR recordAbstract:(+assess*) OR recordAbstract:(+user +experience*) OR recordAbstract:(+efficacy) OR recordAbstract:(+utility) OR recordAbstract:(+utilisation) OR recordAbstract:(+utilization) OR recordAbstract:(+patient* +accept*) OR recordAbstract:(+user* +accept*) OR recordAbstract:(+user* +perce*) OR recordAbstract:(+patient* +perce*) OR recordAbstract:(+user* +perspective) OR recordAbstract:(+patient* +perspective*) OR recordAbstract:(+user* +view*) OR recordAbstract:(+patient* +view*)) | 743 |
