# Supplementary material for: The Effectiveness of Artificial Intelligence Conversational Agents in Health Care: Systematic Review
Source: J Med Internet Res. 2020 Oct 22;22(10):e20346. doi: 10.2196/20346 (PMC7644372; doi:10.2196/20346)
Supplement: Multimedia Appendix 2 [file jmir_v22i10e20346_app2.docx]

### Multimedia Appendix B

The retrieval was conducted in a series of searches due to limitation in the number of search criteria that could be specified. These were conducted in line with the original search categories. The exclusion searches were derived from characteristics of irrelevant studies identified. Pass refers to a search following which the subsequent search was conducted on the subset of studies retrieved in the previous pass.

**First pass:** Any field = Conversation* OR chat* OR virtual OR interactive OR relational OR speech OR voice OR natural language

**Second pass:** Any field = health*

**Third pass:** Any field = Outcome OR evaluat* OR effect* OR efficacy OR feasib* OR usab* OR accepta* OR perce*

**Fourth pass:** Title = NOT (review OR protocol OR guidelines)

**Fifth pass:**  Any field = NOT (surgery OR surgical OR ecol* OR animal OR industr* OR transcription OR imaging OR librar* OR social media)

**Sixth pass:** Year = greater than or equal to 2008

### 
