# Supplementary material for: The Effectiveness of Artificial Intelligence Conversational Agents in Health Care: Systematic Review
Source: J Med Internet Res. 2020 Oct 22;22(10):e20346. doi: 10.2196/20346 (PMC7644372; doi:10.2196/20346)
Supplement: Multimedia Appendix 3 [file jmir_v22i10e20346_app3.docx]

### Multimedia Appendix C. Summary of study characteristics

| **Citation** | **Study design** | **Country of study** | **Study population** | **N (study arms)** | **Conversational agent** |
| --- | --- | --- | --- | --- | --- |
| [9] Adams WG, Phillips BD, Bacic JD, Walsh KE, Shanahan CW, Paasche-Orlow MK. Automated conversation system before pediatric primary care visits: a randomized trial. Pediatrics 2014 Sep;134(3):e691–9. PMID:25092938 | RCT | USA | Children aged 4 months to 11 years who had an RHCM or well-child visit | 475 (Personal Health Partner: n=293; single automated call: n=182) | Personal Health Partner |
| [46] Bibault J-E, Chaix B, Guillemassé A, Cousin S, Escande A, Perrin M, Pienkowski A, Delamon G, Nectoux P, Brouard B. A Chatbot Versus Physicians to Provide Information for Patients With Breast Cancer: Blind, Randomized Controlled Noninferiority Trial. J Med Internet Res 2019 Nov 27;21(11):e15787. PMID:31774408 | RCT | France | Patients with breast cancer and their relatives | 142 (Vik: n=71; physician: n=71) | Vik |
| [50] Borja-Hart NL, Spivey CA, George CM. Use of virtual patient software to assess student confidence and ability in communication skills and virtual patient impression: A mixed-methods approach. Curr Pharm Teach Learn 2019 Jul;11(7):710–718. PMID:31227094 | Pre-post | USA | Pharmacy students | 203 | Shadow Health |
| [32] Cameron G, Cameron D, Megaw G, Bond R, Mulvenna M, O’Neill S, Armour C, McTear M. Assessing the Usability of a Chatbot for Mental Health Care. In: Bodrunova S. et al. Internet Science., editor. Lecture Notes in Computer Science, vol 11551 Springer, Cham; 2019. | Cross-  sectional | UK | Employees from a mental health enterprise | 7 | iHelpr |
| [45] Chaix B, Bibault J-E, Pienkowski A, Delamon G, Guillemassé A, Nectoux P, Brouard B. When Chatbots Meet Patients: One-Year Prospective Study of Conversations Between Patients With Breast Cancer and a Chatbot. JMIR Cancer 2019;5(1):e12856. | Cross-  sectional | France | Patients with breast cancer and their relatives | 958 | Vik |
| [8] Chang P, Sheng Y-H, Sang Y-Y, Wang D-W. Developing a wireless speech- and touch-based intelligent comprehensive triage support system. Comput Inform Nurs 2008 Jan;26(1):31–38. PMID:18091619 | Cross-  sectional | Taiwan | Emergency department patients | 30 | Speech and touch based intelligent comprehensive triage support system |
| [54] Crutzen R, Peters G-JY, Portugal SD, Fisser EM, Grolleman JJ. An artificially intelligent chat agent that answers adolescents’ questions related to sex, drugs, and alcohol: an exploratory study. J Adolesc Health 2011 May;48(5):514–519. PMID:21501812 | Cross-  sectional | The Netherlands | Adolescents | 929 | Bzz |
| [42] Dimeff LA, Jobes DA, Chalker SA, Piehl BM, Duvivier LL, Lok BC, Zalake MS, Chung J, Koerner K. A novel engagement of suicidality in the emergency department: Virtual Collaborative Assessment and Management of Suicidality. Gen Hosp Psychiatry 2018;63:119–126. PMID:29934033 | Cross-  sectional | USA | Emergency department patients admitted due to acute suicidal crisis | 24 | Dr Dave |
| [33] Elmasri D, Maeder A. A Conversational Agent for an Online Mental Health Intervention [Internet]. Brain Informatics and Health. 2016. p. 243–251. [doi: 10.1007/978-3-319-47103-7_24] | Cross-  sectional | Australia | Young adults (18-25) at low to medium risk of alcoholism | 17 | Chatbot to address substance abuse |
| [13] Fitzpatrick KK, Darcy A, Vierhile M. Delivering Cognitive Behavior Therapy to Young Adults With Symptoms of Depression and Anxiety Using a Fully Automated Conversational Agent (Woebot): A Randomized Controlled Trial. JMIR Ment Health 2017 Jun 6;4(2):e19. PMID:28588005 | RCT | USA | University students who self-identified with depression and anxiety | 70 (Woebot: n = 34, e-book control: n = 36) | Woebot |
| [53] Friederichs S, Bolman C, Oenema A, Guyaux J, Lechner L. Motivational Interviewing in a Web-Based Physical Activity Intervention With an Avatar: Randomized Controlled Trial [Internet]. Journal of Medical Internet Research. 2014. p. e48. [doi: 10.2196/jmir.2974] | RCT | The Netherlands | Adults (18-70) | 958; 500 at follow up 2 (avatar: n=162; text: n=146; control: n=192) | AVATAR |
| [34] Fulmer R, Joerin A, Gentile B, Lakerink L, Rauws M. Using Psychological Artificial Intelligence (Tess) to Relieve Symptoms of Depression and Anxiety: Randomized Controlled Trial. JMIR Ment Health 2018 Dec 13;5(4):e64. PMID:30545815 | RCT | USA | Students | 75 (intervention: n=50 [group 1: n=24; group 2: n=26]; control: n=25) | Tess |
| [52] Galescu L, Allen J, Ferguson G, Quinn J, Swift M. Speech recognition in a dialog system for patient health monitoring [Internet]. 2009 IEEE International Conference on Bioinformatics and Biomedicine Workshop. 2009. [doi: 10.1109/bibmw.2009.5332111] | Cross-  sectional | USA | Patients with chronic heart failure | 14 | Computer assistant for robust dialogue interaction and care (CARDIAC) |
| [44] Ghosh S, Bhatia S, Bhatia A. Quro: Facilitating User Symptom Check Using a Personalised Chatbot-Oriented Dialogue System. Stud Health Technol Inform 2018;252:51–56. PMID:30040682 | Qualitative | Australia | Clinical scenarios | 30 | Quro |
| [14] Håvik R, Wake JD, Flobak E, Lundervold A, Guribye F. A Conversational Interface for Self-screening for ADHD in Adults [Internet]. Internet Science. 2019. p. 133–144. [doi: 10.1007/978-3-030-17705-8_12 ] | Cohort | Norway | General population | 11 | ROB |
| [47] Heyworth L, Kleinman K, Oddleifson S, Bernstein L, Frampton J, Lehrer M, Salvato K, Weiss TW, Simon SR, Connelly M. Comparison of interactive voice response, patient mailing, and mailed registry to encourage screening for osteoporosis: a randomized controlled trial. Osteoporos Int 2014 May;25(5):1519–1526. PMID:24566584 | RCT | United States | Women 50-64 with risk factors for osteoporosis | 4685 (IVR call: n = 1565, usual care: n = 1558, usual care + mailed info: n = 1562) | Interactive Voice Response (IVR) phone call |
| [35] Hudlicka E. Virtual training and coaching of health behavior: example from mindfulness meditation training. Patient Educ Couns 2013 Aug;92(2):160–166. PMID:23809167 | Cohort | Not reported | Students | 32 (coach vs. written and audio materials, n not stated) | “Chris” (virtual mindfulness coach) |
| [36] Inkster B, Sarda S, Subramanian V. An Empathy-Driven, Conversational Artificial Intelligence Agent (Wysa) for Digital Mental Well-Being: Real-World Data Evaluation Mixed-Methods Study. JMIR Mhealth Uhealth 2018 Nov 23;6(11):e12106. PMID:30470676 | Cohort | Global | General population | 129 | Wysa app |
| [56] Ireland D, Atay C, Liddle J, Bradford D, Lee H, Rushin O, Mullins T, Angus D, Wiles J, McBride S, Vogel A. Hello Harlie: Enabling Speech Monitoring Through Chat-Bot Conversations. Stud Health Technol Inform 2016;227:55–60. PMID:27440289 | Qualitative | Australia | General population | 33 | Harlie |
| [15] Isaza-Restrepo A, Gómez MT, Cifuentes G, Argüello A. The virtual patient as a learning tool: a mixed quantitative qualitative study [Internet]. BMC Medical Education. 2018. [doi: 10.1186/s12909-018-1395-8 ] | Pre-post | Columbia | Undergraduate medical students | 20 | Virtual Patient |
| [37] Ly KH, Ly A-M, Andersson G. A fully automated conversational agent for promoting mental well-being: A pilot RCT using mixed methods. Internet Interv 2017 Dec;10:39–46. PMID:30135751 | RCT | Sweden | Non-clinical population | 28 (chatbot: n = 14, wait-list control: n = 14) | Shim |
| [12] Nakagawa S, Enomoto D, Yonekura S, Kanazawa H, Kuniyoshi Y. A Telecare System that Estimates Quality of Life through Communication [Internet]. 2018 5th IEEE International Conference on Cloud Computing and Intelligence Systems (CCIS). 2018. [doi: 10.1109/ccis.2018.8691360] | Qualitative | Japan | General population | 14 | Telecare system that estimates QoL |
| [51] Philip P, Bioulac S, Sauteraud A, Chaufton C, Olive J. Could a Virtual Human Be Used to Explore Excessive Daytime Sleepiness in Patients? [Internet]. Presence: Teleoperators and Virtual Environments. 2014. p. 369–376. [doi: 10.1162/pres_a_00197] | Cohort | France | Sleep clinic patients and health controls | 62 (patients: n = 32, controls: n = 30) | Virtual physician (Embodied Conversational Agent) |
| [38] Philip P, Micoulaud-Franchi J-A, Sagaspe P, De Sevin E, Olive J, Bioulac S, Sauteraud A. Virtual human as a new diagnostic tool, a proof of concept study in the field of major depressive disorders [Internet]. Scientific Reports. 2017. [doi: 10.1038/srep42656] | Cluster crossover | France | Sleep clinic patients aged 18-65 | 179 | ECA for diagnosing MDD |
| [48] Rhee H, Allen J, Mammen J, Swift M. Mobile phone-based asthma self-management aid for adolescents (mASMAA): a feasibility study. Patient Prefer Adherence 2014 Jan 7;8:63–72. PMID:24470755 | Qualitative | United States | Adolescent-parent dyads | 15 dyads | Mobile phone-based asthma self-management aid for adolescents (mASMAA) |
| [49] Simon SR, Zhang F, Soumerai SB, Ensroth A, Bernstein L, Fletcher RH, Ross-Degnan D. Failure of automated telephone outreach with speech recognition to improve colorectal cancer screening: a randomized controlled trial. Arch Intern Med 2010 Feb 8;170(3):264–270. PMID:20142572 | RCT | United States | Men and women aged 50 to 64 | 20,938 (ATO-SR: n = 10,432, usual care: n = 10,506) | Automated telephone outreach with speech recognition (ATO-SR) |
| [43] Spänig S, Emberger-Klein A, Sowa J-P, Canbay A, Menrad K, Heider D. The virtual doctor: An interactive clinical-decision-support system based on deep learning for non-invasive prediction of diabetes. Artif Intell Med 2019 Sep;100:101706. PMID:31607340 | Prospective, cross-  sectional | Germany | University students | 320 | Virtual doctor |
| [41] Washburn M, Bordnick P, Rizzo AS. A pilot feasibility study of virtual patient simulation to enhance social work students’ brief mental health assessment skills. Soc Work Health Care 2016 Oct;55(9):675–693. PMID:27552646 | Qualitative | United States | Medical students | 5 | Virtual patient software |
| [55] Wong W, Thangarajah J, Padgham L. Contextual question answering for the health domain [Internet]. Journal of the American Society for Information Science and Technology. 2012. p. 2313–2327. [doi: 10.1002/asi.22733 ] | Qualitative | Australia | N/A | N/A | enquireMe |
| [40] Xu R, Mei G, Zhang G, Gao P, Judkins T, Cannizzaro M, Li J. A voice-based automated system for PTSD screening and monitoring. Stud Health Technol Inform 2012;173:552–558. PMID:22357057 | Qualitative | None | Voice clips of US soldiers | 10 | Tele-PTSD Monitor |
| [39] Yasavur U, Lisetti C, Rishe N. Let’s talk! speaking virtual counselor offers you a brief intervention. Journal on Multimodal User Interfaces 2014;8:381–398. | Cross-  sectional | United States | University students | 89 (52 training system, 37 testing system) | Virtual alcohol counsellor (ECA) |
